# Green Extraction and Molecular Docking-Guided Discovery of Asiaticoside as a Multi-Target Modulator of the SIRT1/HMGB1/p53-p21 Axis in H_2_O_2_-Induced Senescent Endothelial Cells

**DOI:** 10.3390/molecules31193451

**Published:** 2026-09-28

**Authors:** Wenkai Li, Jianhua Huang, Fei Zeng, Yuping Wang, Xiong Liu, Junjie Wang, Siyu Liu

**Affiliations:** 1 School of Pharmacy, Xiangnan University, Chenzhou 423000, China; liwenkai1990@126.com (W.L.); hjh530468526@163.com (J.H.); zengfei@iccas.ac.cn (F.Z.); 3119020091@stu.cpu.edu.cn (Y.W.); liuxiong250209@163.com (X.L.); 2 Institute of Industrial Technology, Xiangnan University, Chenzhou 423000, China; xiangnyx@163.com

**Keywords:** asiaticoside, endothelial senescence, SIRT1, HMGB1, molecular docking, green extraction, SASP, HUVECs

## Abstract

Endothelial cell senescence is a key contributor to vascular ageing and cardiovascular diseases. Asiaticoside, a pentacyclic triterpenoid saponin from *Centella asiatica*, exhibits anti-inflammatory and antioxidant activities, yet its potential to modulate endothelial senescence remains unexplored. Herein, we report the molecular docking-guided discovery of asiaticoside as a multi-target modulator of the SIRT1/HMGB1/p53–p21 senescence regulatory axis. Asiaticoside was isolated via a green acid-hydrolysis strategy using dilute HCl as the extraction solvent, followed by polyamide enrichment and C_18_ reversed-phase purification (purity > 98%). Molecular docking revealed that asiaticoside binds favourably to SIRT1 (−8.1 kcal mol^−1^), the HMGB1–DNA complex (−10.7 kcal mol^−1^), and several senescence-associated secretory phenotype (SASP)-related inflammatory cytokines. In H_2_O_2_-induced senescent human umbilical vein endothelial cells (HUVECs), asiaticoside at 100 μM significantly reduced senescence-associated β-galactosidase activity and alleviated G0/G1 cell cycle arrest without cytotoxicity. Mechanistically, asiaticoside restored SIRT1 expression, suppressed the secretion of IL-1β, IL-6, IL-8, TNF-α, and HMGB1, downregulated p21 at both mRNA and protein levels, reduced p53 protein expression, and suppressed p16 mRNA expression. These findings demonstrate that asiaticoside attenuates endothelial senescence and SASP-driven inflammation, an effect associated with restoration of SIRT1 expression and suppression of the HMGB1/p53–p21 pathway.

## 1. Introduction

Cardiovascular and cerebrovascular diseases remain the leading causes of morbidity and mortality in the ageing population, and delaying vascular ageing has emerged as a promising strategy for their prevention [1]. Vascular ageing is accelerated by atherosclerosis, type 2 diabetes, hypertension, and stroke [2]. At the cellular level, endothelial cells (ECs) play a central role in maintaining vascular homeostasis. Endothelial senescence, a state of irreversible growth arrest accompanied by phenotypic dysfunction, is a fundamental contributor to vascular ageing [3]. Oxidative stress, particularly hydrogen peroxide (H_2_O_2_), is a well-characterised inducer of stress-induced premature senescence (SIPS) [4,5]. Senescent ECs exhibit elevated senescence-associated β-galactosidase (SA-β-gal) activity, G_1_/S cell cycle arrest, and a senescence-associated secretory phenotype (SASP) [6,7]. These findings underscore the critical involvement of EC senescence in vascular pathologies, yet the molecular mechanisms linking oxidative stress to endothelial senescence remain incompletely understood [8,9,10].

Chronic inflammation is both a driver and an amplifier of cellular senescence. Pro-inflammatory cytokines, including interleukin-6 (IL-6), interleukin-1β (IL-1β), and tumour necrosis factor-α (TNF-α), induce or accelerate senescence in various cell types through p53/p21- and nuclear factor-κB (NF-κB)-dependent pathways [11,12,13]. In the vasculature, persistent low-grade inflammation directly triggers EC senescence [14,15]. High mobility group box 1 (HMGB1) is a nuclear architectural factor that, upon extracellular release, acts as a damage-associated molecular pattern (DAMP) and perpetuates sterile inflammation [16]. Extracellular HMGB1 engages pattern-recognition receptors such as RAGE and TLR4, establishing a self-amplifying inflammatory loop [17]. Oxidative stress promotes the acetylation-dependent translocation and release of HMGB1, linking ROS to innate immune activation [18,19]. We previously showed that HMGB1 expression was elevated in the aorta of type 2 diabetic rats and that exogenous HMGB1 increased SA-β-gal activity in cultured HUVECs [20]. These observations suggest that HMGB1 may bridge oxidative stress and endothelial senescence; however, the downstream mechanism remains unclear.

Sirtuin 1 (SIRT1) is a nicotinamide adenine dinucleotide (NAD^+^)-dependent class III histone deacetylase that is highly expressed in endothelial cells and regarded as a key longevity regulator [21,22]. SIRT1 regulates inflammation, oxidative stress defence, and cellular senescence primarily through deacetylation of key substrates [23]. SIRT1 deacetylates p53, thereby attenuating p53-mediated activation of p21 and preventing premature senescence [24,25]. Age-related decline in endothelial SIRT1 expression accelerates cellular senescence [26,27]. SIRT1 also suppresses NF-κB-driven inflammation and inhibits HMGB1 release, suggesting a negative regulatory relationship between SIRT1 and the HMGB1-inflammation axis [28,29]. However, whether HMGB1 reciprocally suppresses SIRT1 expression in endothelial cells, thereby creating a feed-forward loop that amplifies inflammatory senescence, has not been investigated. We hypothesise that HMGB1 promotes endothelial senescence, at least in part, by suppressing SIRT1 and relieving the deacetylation-mediated restraint on p53.

Asiaticoside is a principal triterpenoid saponin of *Centella asiatica* (L.) Urban, a plant widely used in traditional Chinese and Ayurvedic medicine for wound healing, anti-inflammation, and other bioactivities [30,31,32,33,34,35]. Notably, *C. asiatica* is recognised not only as a medicinal herb but also as an edible plant, reflecting the concept of “medicine-food homology” that has attracted increasing regulatory and scientific attention [36,37]. From this perspective, food-derived functional ingredients are of growing interest for healthy ageing [38]. Pharmacological studies have shown that asiaticoside upregulates SIRT1 and protects against renal ischaemia–reperfusion injury [35], and suppresses LPS-induced production of inflammatory cytokines [33]. Nevertheless, the potential role of asiaticoside in endothelial senescence remains unexplored.

In the present study, we first established a green and scalable extraction protocol for asiaticoside using dilute HCl as the primary extraction solvent, thereby substantially reducing the reliance on organic solvents compared with conventional ethanol- or methanol-based approaches. We then investigated, to the best of our knowledge for the first time, the protective effect of asiaticoside against H_2_O_2_-induced premature senescence in HUVECs and explored the underlying molecular mechanism. We hypothesised that H_2_O_2_-induced oxidative stress triggers HMGB1 release and downstream inflammatory signalling, which in turn suppress SIRT1 expression, leading to p53 accumulation, p21 upregulation, and the establishment of a senescent phenotype, and that asiaticoside counteracts this cascade by restoring SIRT1 expression and suppressing HMGB1-mediated inflammatory signalling. To test this hypothesis, we integrated molecular docking, cell-based senescence assays (SA-β-gal staining, cell cycle analysis, and MTT viability), enzyme-linked immunosorbent assay (ELISA), quantitative real-time polymerase chain reaction (qPCR), and Western blot analysis.

## 2. Results and Discussion

### 2.1. Green Extraction, Enrichment and Purification of Asiaticoside from Centella asiatica

The chemical structure of asiaticoside, a pentacyclic triterpenoid saponin, is shown in Figure 1A. The isolation and purification of asiaticoside from the *C. asiatica* extract were monitored throughout by HPLC, which provided rapid and reliable tracking of the target compound during extraction and chromatographic fractionation. The HPLC chromatogram of the extract obtained under the optimised conditions displayed a major peak with a retention time identical to that of the asiaticoside standard (Figure 1B,C), indicating satisfactory specificity.

To maximise the extraction efficiency, four key parameters were initially evaluated through single-factor experiments. The hydrochloric acid concentration exerted the most pronounced influence on the yield of asiaticoside (Figure 2A). The peak area increased sharply as the HCl concentration rose from 0.01 to 0.02 mol L^−1^, reaching a maximum at 0.02 mol L^−1^, and then decreased progressively at higher concentrations (0.03–0.08 mol L^−1^). This trend indicates that a mildly acidic environment facilitates the release of asiaticoside from the plant matrix, presumably by promoting the hydrolysis of cell wall polysaccharides and enhancing the solubility of the triterpenoid saponin; however, excessive acidity likely causes cleavage of glycosidic bonds, resulting in degradation of the target compound. Regarding the solid–liquid ratio (Figure 2B), the peak area increased markedly as the ratio varied from 1:5 to 1:15, after which the improvement plateaued. A ratio of 1:15 was therefore chosen as the optimal balance between extraction efficiency and solvent consumption. The extraction time study (Figure 2C) showed that the asiaticoside yield reached its maximum at 2 h, and a slight decline was observed when the extraction was prolonged to 3 h, possibly due to thermal instability of the saponin under prolonged reflux. Finally, two extraction cycles were found to be sufficient; the third cycle yielded only a negligible amount of asiaticoside, as detected by HPLC. Consequently, the preliminary optimal conditions identified from the single-factor assays were: HCl concentration 0.02 mol L^−1^, solid–liquid ratio 1:15, extraction time 2 h per cycle, and two extraction cycles.

An L_9_(3^3^) orthogonal array experiment was subsequently designed to further refine the extraction conditions, based on the factors and levels listed in Table 1. The experimental results and range analysis are presented in Table 2. The k values (Figure 2D) and the calculated range (R, Figure 2E) demonstrate that the influence of the three factors on the asiaticoside peak area decreased in the order: HCl concentration (A, R = 865.80) > solid–liquid ratio (B, R = 287.85) > extraction time (C, R = 272.17). HCl concentration was thus identified as the dominant factor. The maximum k_2_ values for factors A and B and k_3_ for factor C indicated that the intuitively optimal combination would be A_2_B_2_C_3_, i.e., 0.02 mol L^−1^ HCl, a solid–liquid ratio of 1:15, and an extraction time of 3 h. However, analysis of variance (Table 3) revealed that none of the three factors reached statistical significance, although factor A displayed the largest F value (F = 13.68, *p* = 0.068). The lack of statistical significance is likely attributable to the limited degrees of freedom resulting from the absence of replicate runs in the orthogonal design. Importantly, the difference in the *k* values between C_2_ (2 h) and C_3_ (3 h) was marginal (<2% relative difference), and the single-factor experiments had shown a declining trend after 2 h. Taking into account both process economy and the single-factor data, the final optimised conditions were set as A_2_B_2_C_2_: HCl concentration 0.02 mol L^−1^, solid–liquid ratio 1:15, and extraction twice for 2 h each.

To verify the reliability of the optimised procedure, a scale-up experiment was carried out with a 20-fold increase in the amount of starting material (60.0 g). Under these conditions, the extracted asiaticoside content in the dried extract was 2.84% (*w*/*w*), and the extraction recovery was 70.17% (based on the initial asiaticoside content of the raw material), with a relative standard deviation of less than 3% in triplicate experiments. This confirms the stability and reproducibility of the acid-based extraction method. It should be noted that the orthogonal design was performed without replicates; therefore, the statistical significance of the ANOVA is limited. This design served only as a preliminary guide to identify the approximate levels of the three factors. The final optimised conditions were confirmed by subsequent scale-up validation experiments (performed in triplicate, RSD < 3%), which, together with the consistent trends observed in the single-factor experiments, provide practical confidence in the robustness and reproducibility of the extraction protocol.

The crude extract was subjected to polyamide column chromatography for enrichment. The majority of pigments (particularly chlorophylls) and polysaccharides were retained on the column, while asiaticoside was eluted in the 30% ethanol fraction. HPLC analysis showed that the asiaticoside content in the pooled 30% ethanol eluate increased to 83.65% in the initial elution. A repeated enrichment run yielded an asiaticoside content of 54.03%, with the lower value likely attributable to progressive fouling of the polyamide resin upon reuse. This step effectively removed highly polar impurities and significantly concentrated the target compound.

Final purification was achieved using a UniSil 30–120 C_18_ reversed-phase silica gel column with a stepwise methanol–water gradient. HPLC monitoring indicated that asiaticoside was exclusively eluted with 50% methanol. HPLC profiles demonstrated that asiaticoside reached its highest purity of 98.38% in the 10th bed volume (BV) during the gradient elution. In a validation run under the optimised conditions, the purity of asiaticoside was further improved to 98.65%. Starting from an initial content of 2.35% in the raw plant material, this represents a substantial enrichment of over 40-fold.

The purified asiaticoside was obtained as a white amorphous powder. Its molecular formula was determined to be C_48_H_78_O_19_ by HR-ESI-MS, based on the [M + Na]^+^ ion at *m*/*z* 981.5035 (calcd for 981.5029) in positive-ion mode and the [M + HCOO]^−^ ion at *m*/*z* 1003.5116 (calcd for C_48_H_78_O_19_HCOO^−^, 1003.5113) in negative-ion mode (Figure 1D). The ^1^H NMR spectrum (Figure 1E) displayed six methyl singlets at *δ*_H_ 0.72–1.16, an olefinic proton at δH 5.27 (1H, t, *J* = 3.5 Hz), and three anomeric protons at *δ*_H_ 5.31 (1H, d, *J* = 8.2 Hz), 4.86 (1H, d, *J* = 1.3 Hz), and 4.39 (1H, d, *J* = 7.9 Hz), indicating the presence of two glucose units and one rhamnose unit. The ^13^C NMR spectrum (Figure 1F) exhibited 48 carbon resonances, including three anomeric carbons at *δ*_C_ 94.44, 103.08, and 101.49, along with characteristic olefinic carbons at *δ*_C_ 137.97 (C-13) and 125.55 (C-12) and an ester carbonyl at δC 176.58 (C-28). These spectroscopic data were in complete agreement with those reported for asiaticoside [39], confirming both the identity and high purity of the isolated compound.

The integrated “acid hydrolysis extraction–polyamide enrichment–C_18_ reversed-phase purification” strategy established in this work offers several advantages. First, the acid-based extraction method uses only a very dilute HCl solution (0.02 mol L^−1^) as the extraction solvent, drastically reducing the consumption of organic solvents compared with conventional ethanol- or methanol-based extraction protocols [40,41]. The resulting extract is also noticeably lighter in colour, as fewer lipophilic pigments are co-extracted, which simplifies downstream purification. Second, the combination of polyamide and C_18_ stationary phases provides a complementary separation mechanism: the polyamide resin effectively removes highly polar impurities, while the C_18_ column resolves asiaticoside from other structurally similar components based on subtle differences in hydrophobicity. This tandem approach delivered asiaticoside with a purity exceeding 98%, which meets the stringent purity requirements for downstream applications. Similar orthogonal design approaches have been widely employed for the optimisation of natural product extraction, and the results are consistent with the general recognition that acid concentration is often the predominant factor in the extraction of triterpenoid saponins. The entire process is operationally simple, economically viable, and readily scalable, providing a solid foundation for the further development and quality control of *C. asiatica* saponins.

The high-purity asiaticoside obtained through this green and scalable process was used for all subsequent cell-based biological assays. In parallel, the structure of asiaticoside was used for molecular docking simulations to predict its interactions with senescence- and inflammation-related targets.

### 2.2. Molecular Docking Reveals Direct Binding Potential of Asiaticoside to Key Senescence and Inflammation Targets

Following the structural confirmation of asiaticoside (Section 2.1), molecular docking simulations based on its chemical structure were performed against seven targets implicated in endothelial senescence and inflammation—SIRT1, the HMGB1–DNA complex, TNF-α, IL-1β, IL-6, p53, and p21—to generate testable hypotheses for the subsequent cellular experiments. The predicted binding affinities, calculated by AutoDock Vina [42], ranged from −10.7 to −5.1 kcal mol^−1^ (Figure 3A). Asiaticoside displayed moderate-to-strong predicted affinity toward SIRT1 (−8.1 kcal mol^−1^) and the HMGB1–DNA complex (−10.7 kcal mol^−1^), moderate affinity toward TNF-α (−7.3 kcal mol^−1^), IL-1β (−7.5 kcal mol^−1^), and IL-6 (−6.5 kcal mol^−1^), and modest affinity toward p53 (−7.2 kcal mol^−1^) and p21 (−5.1 kcal mol^−1^). Notably, the scoring function baselines differ between protein–ligand and nucleic acid–ligand systems; therefore, the value for the HMGB1–DNA complex should be interpreted with caution and cannot be directly compared with the protein-only targets. Nonetheless, the consistently negative binding energies across all seven targets suggest that asiaticoside possesses a polypharmacological binding profile that may simultaneously engage multiple nodes of the senescence regulatory network.

In the SIRT1 catalytic domain, asiaticoside adopted an extended conformation occupying the cleft that normally accommodates NAD^+^ and the acetyl-lysine substrate (Figure 3B). Four hydrogen bonds were formed with three residues: the side chain of Gln361 (two bonds, 2.4–2.8 Å), the side chain of Glu410 (2.8 Å), and the backbone N–H of Ile359 (1.8 Å). The rigid pentacyclic triterpene backbone was anchored through van der Waals contacts with the aliphatic side chain of Ile359 and surrounding hydrophobic residues, while the oligosaccharide moiety oriented toward the mouth of the pocket near Glu410. Gln361 is positioned immediately adjacent to the conserved catalytic histidine His363, and Glu410 lies within the zinc-binding subdomain of SIRT1 [43]. This binding mode, in which the ligand occupies the catalytic cleft and interacts with substrate-adjacent residues, is consistent with a potential substrate-competitive interaction. These docking results predict that asiaticoside may bind with the SIRT1 catalytic domain, providing a structural hypothesis for the upregulation of SIRT1 expression observed in subsequent cellular experiments (Section 2.4). However, the predicted binding site lies within the substrate-binding cleft, which is more consistent with a substrate-competitive interaction than with allosteric activation. Therefore, the docking results alone do not explain the observed upregulation of SIRT1 protein expression.

For the HMGB1–DNA complex, asiaticoside inserted into the widened minor-groove region was recognised by the HMG-box domains (Figure 3C). Remarkably, nine hydrogen bonds were formed with five nucleotide residues of the DNA duplex—dT4, dC5, dG6, dA13, and dC15—rather than with protein side chains. The donor–acceptor distances (2.1–2.8 Å) indicated strong polar anchoring of the ligand within the minor groove. This DNA-centric binding mode suggests that asiaticoside may competitively interfere with HMGB1–DNA recognition, a process required for the architectural functions of HMGB1 in chromatin and for its release upon cellular stress. Given that extracellular HMGB1 is a potent DAMP that perpetuates SASP signalling, direct sequestration at the DNA interface could represent a potential additional mechanism to limit its pro-inflammatory activity [44]. This DNA-centric binding prediction has not been experimentally validated by biochemical assays such as electrophoretic mobility shift assay (EMSA) or surface plasmon resonance (SPR); therefore, its functional relevance remains to be established.

The docking poses against the inflammatory cytokines revealed a conserved pattern: asiaticoside bound to surface grooves or subunit-interface clefts that are critical for receptor engagement (Figure 3D–F). In TNF-α, the ligand occupied the subunit-interface cleft, forming eight hydrogen bonds with residues from two adjacent subunits (Asp143, Leu142, Ser65, Gln67, Cys69; distances 2.2–3.1 Å). This region is essential for trimer stability and TNFR recognition [45]. In IL-1β, asiaticoside engaged the surface-exposed Glu64–Lys65 loop within the β-trefoil fold (four hydrogen bonds, 2.2–2.5 Å), a charged region that directly participates in IL-1R1 binding and assembly of the signalling complex [46]. In IL-6, the ligand nestled into an interhelical groove of the four-helix-bundle fold, contacting Lys120 (helix C, gp130-binding site II) and Asn144 (CD loop, adjacent to site III) via three hydrogen bonds (2.1–2.3 Å). These interactions are predicted to disrupt the sequential recruitment of gp130, thereby interfering with hexameric signalling complex assembly [47]. Collectively, these binding modes suggest that asiaticoside might function as a predicted neutralising ligand for multiple SASP components by competitively occupying receptor-binding surfaces.

Asiaticoside also demonstrated the ability to engage the DNA-binding domain of p53, a master transcriptional activator of p21 (Figure 3G). The ligand bound within a surface groove that is critical for sequence-specific DNA recognition, forming five hydrogen bonds with Leu111, Phe113, His115, and Asn131 (heavy-atom distances 2.3–3.2 Å; His115 H··· acceptor distance 1.8 Å). This region includes the L1 loop that directly contacts the DNA backbone in the p53–DNA complex [48]. By occupying this groove, asiaticoside may sterically hinder p53–DNA binding and consequently reduce p53-mediated transactivation of CDKN1A (p21). Notably, this direct binding to p53 is complementary to the SIRT1-mediated deacetylation and destabilisation of p53 protein observed in our cellular experiments (Section 2.4), suggesting a potential dual-level mechanism for p53 pathway suppression. Finally, docking to p21 yielded the least favourable binding energy (−5.1 kcal mol^−1^) among all targets. The ligand associated with the basic C-terminal segment (Lys154–Arg155) adjacent to the PCNA-interacting motif (Figure 3H), forming four hydrogen bonds (2.1–2.6 Å). While the modest predicted affinity suggests that direct p21 binding may only serve an auxiliary role, it could contribute to the overall cell cycle-restoring effect by partially interfering with p21–PCNA interactions [49].

Taken together, the molecular docking results predict that asiaticoside may bind to the catalytic cleft of SIRT1, the DNA interface of HMGB1, the receptor-engaging surfaces of TNF-α, IL-1β, and IL-6, and the functional domains of p53 and p21. This computational profile provides structural hypotheses for the multi-target effects observed in subsequent cellular experiments. However, it should be emphasised that molecular docking provides computational predictions of binding modes and affinities, and none of the predicted interactions were validated by direct biophysical techniques such as surface plasmon resonance (SPR), cellular thermal shift assay (CETSA), or electrophoretic mobility shift assay (EMSA). Consequently, the observed changes in expression levels cannot be directly attributed to the predicted binding events.

### 2.3. Asiaticoside Attenuates H_2_O_2_-Induced Senescence and Cell Cycle Arrest in HUVECs

Based on the molecular docking evidence that asiaticoside could potentially interact with key regulatory nodes of cellular senescence and inflammation, we proceeded to evaluate its protective effects in an H_2_O_2_-induced premature senescence model of HUVECs. The cytotoxicity of asiaticoside was first assessed by the MTT assay. As shown in Figure 4A, treatment with asiaticoside at concentrations ranging from 10 to 100 μM for 24 h did not significantly affect HUVEC viability compared with the vehicle control (*p* > 0.05), confirming the biosafety of the tested concentrations and excluding non-specific cytotoxicity as a confounding factor in the subsequent functional assays.

We next examined whether asiaticoside could protect HUVECs from H_2_O_2_-induced cellular senescence using SA-β-gal staining. Exposure to 400 μM H_2_O_2_ for 4 h followed by 24 h recovery resulted in a marked increase in the percentage of SA-β-gal-positive cells (*p* < 0.01 vs. control, Figure 4B,C), confirming the successful establishment of the premature senescence model. Pretreatment with asiaticoside significantly reduced the senescent cell population in a dose-dependent manner, with the 100 μM concentration exhibiting the most pronounced anti-senescence effect. The positive control resveratrol (10 μM) also effectively attenuated senescence (*p* < 0.01 vs. model), validating the responsiveness of the model to a known SIRT1 activator. Notably, asiaticoside at 100 μM outperformed resveratrol and both lower asiaticoside concentrations while maintaining a cytotoxicity profile indistinguishable from the vehicle control. This favourable combination of superior anti-senescence efficacy and minimal cytotoxicity established 100 μM as the optimal concentration for subsequent cell cycle and mechanistic studies.

Endothelial cell senescence is not merely a hallmark of vascular ageing but also a critical contributor to the initiation and progression of cardiovascular and cerebrovascular diseases, including atherosclerosis, hypertension, and ischemic stroke [50]. A defining feature of senescent endothelial cells is irreversible cell cycle arrest, predominantly at the G1/S transition, which compromises the regenerative capacity of the endothelium and promotes a pro-inflammatory, pro-thrombotic vascular phenotype [51]. Therefore, therapeutic strategies aimed at preserving endothelial proliferative capacity by preventing senescence-associated cell cycle arrest hold considerable promise for cardiovascular protection [52]. To determine whether asiaticoside could alleviate H_2_O_2_-induced cell cycle perturbations, we performed flow cytometric analysis of propidium iodide-stained cells. As illustrated in Figure 4D,E, H_2_O_2_ exposure led to a significant accumulation of cells in the G0/G1 phase (*p* < 0.05 vs. control), accompanied by a marked reduction in the G2/M population (*p* < 0.01 vs. control), a distribution pattern indicative of senescence-associated growth arrest. Pretreatment with 100 μM asiaticoside significantly reversed these alterations: the G0/G1 fraction was reduced (*p* < 0.05 vs. model) and the G2/M population was restored to a level comparable to that of the control group (*p* < 0.01 vs. model). The S-phase population was also significantly increased in the model group (*p* < 0.05 vs. control) and restored by asiaticoside treatment (*p* < 0.05 vs. model). These results demonstrate that asiaticoside effectively rescues HUVECs from H_2_O_2_-induced cell cycle arrest, thereby preserving their proliferative potential.

Taken together, these results demonstrate that asiaticoside effectively protects HUVECs from H_2_O_2_-induced premature senescence and cell cycle arrest, with 100 μM identified as the optimal concentration that combines maximal efficacy with minimal cytotoxicity. Given the molecular docking evidence suggesting the potential engagement of SIRT1 and related targets (Section 2.2), we hypothesised that the anti-senescence and cell cycle-restoring effects of asiaticoside are mediated through the SIRT1 signalling network. This hypothesis was further investigated in the following experiments.

### 2.4. Asiaticoside Suppresses the SASP and Modulates the SIRT1/HMGB1/p53-p21 Axis in Senescent HUVECs

To investigate the effect of asiaticoside on the senescence-associated secretory phenotype (SASP), a key feature of cellular senescence that drives chronic vascular inflammation [53], the levels of five SASP-related factors in culture supernatants were measured by ELISA. As shown in Figure 5A, exposure to 400 μM H_2_O_2_ significantly elevated the secretion of IL-1β, IL-6, IL-8, TNF-α, and HMGB1 compared with the control group (** *p* < 0.01). Pretreatment with 100 μM asiaticoside significantly suppressed the H_2_O_2_-induced elevation of all five factors (* *p* < 0.01 vs. model), with levels approaching those of the control group. The coordinate reduction in HMGB1 alongside the other pro-inflammatory cytokines is notable, as extracellular HMGB1 functions as a damage-associated molecular pattern (DAMP) that can engage TLR4 and RAGE to sustain NF-κB-driven cytokine production, thereby amplifying the SASP in a feed-forward manner [17,54]. These results suggest that asiaticoside may attenuate the SASP by targeting upstream regulatory mechanisms rather than individual cytokine pathways.

To determine whether the suppression of SASP factor secretion was accompanied by transcriptional and translational changes in the core senescence regulatory network, qPCR and Western blot analyses were performed. As shown in Figure 5C, H_2_O_2_ exposure significantly upregulated the mRNA expression of IL1B, TNF, HMGB1, CDKN1A (encoding p21), and CDKN2A (encoding p16) (** *p* < 0.01 vs. control), while SIRT1 mRNA expression was markedly suppressed (* *p* < 0.05 vs. control). Treatment with 100 μM asiaticoside significantly reversed all these changes, restoring SIRT1 expression and suppressing the pro-inflammatory and senescence-associated transcripts. At the protein level (Figure 5D,E), H_2_O_2_ treatment significantly decreased SIRT1 while elevating HMGB1, p53, and p21 (** *p* < 0.01 vs. control), and asiaticoside treatment restored all four proteins towards control levels (** *p* < 0.01 vs. model). Of note, p53 protein was upregulated in the model group despite the absence of p53 mRNA data in our qPCR panel. This observation is consistent with the established mechanism whereby oxidative stress stabilises p53 protein through post-translational modifications, including phosphorylation by ATM/ATR kinases and acetylation, rather than through transcriptional induction [24]. SIRT1 has been shown to deacetylate p53, attenuating its transcriptional activity and potentially facilitating its degradation [55]; the downregulation of SIRT1 under oxidative stress may therefore contribute to p53 accumulation. The restoration of SIRT1 expression by asiaticoside, together with the observed reduction in p53 protein, is consistent with this post-translational regulatory mechanism, although direct acetylation assays were not performed in the present study.

The parallel changes at the mRNA and protein levels for SIRT1, HMGB1, and p21 suggest transcriptional regulation, whereas the regulation of p53 appears to be predominantly post-translational. Mechanistically, SIRT1 deacetylates p53 at key lysine residues (e.g., Lys382), attenuating its transcriptional activity and reducing the expression of p21, a cyclin-dependent kinase inhibitor that enforces G_1_/S arrest during senescence [51]. Under conditions of SIRT1 downregulation, hyperacetylated p53 transactivates the CDKN1A promoter, leading to p21 accumulation, inhibition of cyclin E/CDK2 and cyclin D/CDK4/6 complexes, and G_1_-phase arrest [51]. Our Western blot data showing that asiaticoside reduced p53 and p21 protein levels, together with the restoration of cell cycle distribution (Figure 4D,E), are consistent with the relief of this p53/p21-imposed G_1_/S blockade.

In addition to the p53-p21 pathway, the p16–pRb pathway represents an independent senescence checkpoint. p16 (encoded by CDKN2A) inhibits CDK4/6, preventing the phosphorylation of retinoblastoma protein (pRb) and thereby blocking E2F-mediated transcription of S-phase genes [56]. The coordinate upregulation of both CDKN1A and CDKN2A mRNA observed in the present study indicates that H_2_O_2_-induced senescence engages both p53-dependent and p53-independent cell cycle arrest mechanisms. The suppression of CDKN2A mRNA by asiaticoside further suggests that its anti-senescence effect extends to the p16–pRb pathway, although p16 protein levels were not assessed and this conclusion remains to be confirmed at the translational level.

Regarding the HMGB1 axis, SIRT1 has been implicated in regulating the acetylation status and subcellular localisation of HMGB1 [52]. Under conditions of SIRT1 downregulation, HMGB1 may undergo hyperacetylation, translocate from the nucleus to the cytoplasm, and be released extracellularly, where it functions as a DAMP engaging TLR4 and RAGE to propagate NF-κB-driven inflammatory signalling [17,29]. In the present study, ELISA demonstrated elevated extracellular HMGB1 in the model group, while qPCR and Western blot showed increased intracellular HMGB1 expression, indicating enhanced production and release. Asiaticoside treatment simultaneously reduced both intracellular and extracellular HMGB1 levels, consistent with suppressed HMGB1 expression and potentially enhanced nuclear retention, although direct subcellular fractionation and acetylation assays were not performed.

The convergence of computational and experimental findings provides a coherent mechanistic framework for the protective effects of asiaticoside. Molecular docking (Section 2.2) predicted that asiaticoside can interact with SIRT1, HMGB1, p53, and p21, and the expression analyses presented herein provide indirect support for these predictions at the expression level. The restoration of SIRT1 expression by asiaticoside is associated with the suppression of p53/p21- and p16-mediated cell cycle arrest as well as HMGB1-driven SASP amplification. However, several limitations should be acknowledged. First, the present study did not include SIRT1 deacetylase activity assays; therefore, whether asiaticoside directly enhances SIRT1 enzymatic activity remains to be determined. The predicted binding mode at the SIRT1 catalytic cleft is consistent with a substrate-competitive interaction rather than allosteric activation, and the observed upregulation of SIRT1 protein expression may involve transcriptional or post-translational mechanisms that are independent of direct binding. Second, the direct binding of asiaticoside to p53, TNF-α, IL-1β, and IL-6 predicted by molecular docking was not experimentally validated by biophysical techniques such as SPR, CETSA, or co-immunoprecipitation; therefore, the functional relevance of these predicted interactions remains to be established. Third, no SIRT1 or HMGB1 knockdown/overexpression experiments were performed, so the causal role of the SIRT1/HMGB1 axis in the observed anti-senescence effects cannot be definitively concluded. Fourth, p53 and HMGB1 acetylation levels were not directly measured, and the proposed deacetylation mechanisms are inferred from the literature. Fifth, the orthogonal extraction optimisation lacked replicate runs, which limited the statistical confidence of the factor analysis. Finally, the findings are limited to an in vitro H_2_O_2_-induced senescence model, and validation in animal models of vascular ageing is warranted.

## 3. Materials and Methods

### 3.1. Plant Material and Chemicals

Whole plants of *Centella asiatica* (L.) *Urban* were collected in September 2020 from Suxian District, Chenzhou City, Hunan Province, China, and authenticated by Professor Junjie Wang (School of Pharmacy, Xiangnan University). A voucher specimen (No. YP20200920) was deposited in the Pharmaceutical Laboratory of Xiangnan University. The air-dried material was ground to a greyish-green powder. The initial asiaticoside content of the powder was determined to be 2.35% (dry weight basis) by HPLC.

Asiaticoside reference standard (purity > 98%) was purchased from Chengdu Puruifa Science and Technology Development Co., Ltd. (Chengdu, China). HPLC-grade methanol and acetonitrile were obtained from Thermo Fisher Scientific (Waltham, MA, USA). Analytical-grade hydrochloric acid and ethanol were from Hunan Huihong Reagent Co., Ltd. (Changsha, China); ethyl acetate and sodium hydroxide were from Tianjin Damao Chemical Reagent Factory (Tianjin, China); sulfuric acid and acetic acid were from Sinopharm Chemical Reagent Co., Ltd. (Shanghai, China). Polyamide (30–60 mesh) was from Sinopharm Chemical Reagent Co., Ltd. UniSil30–120 C_18_ reversed-phase silica gel (30–120 μm) was from Suzhou Nanomicro Technology Co., Ltd. (Suzhou, China). Resveratrol (≥99%) was from Sigma-Aldrich (St. Louis, MO, USA). Hydrogen peroxide (H_2_O_2_, 30% *w*/*w*) and DMSO (cell culture grade) were from Sigma-Aldrich. Endothelial cell medium (ECM, cat. no. 1001) was from ScienCell Research Laboratories (Carlsbad, CA, USA). Foetal bovine serum (FBS, cat. no. 10099-141) was from Gibco (Thermo Fisher Scientific). Penicillin–streptomycin solution (cat. no. 15140-122) and 0.25% trypsin without EDTA (cat. no. 15050-065) were from Gibco. Purified water was prepared using a Milli-Q Integral system (Merck Millipore, Burlington, MA, USA).

### 3.2. Optimisation of Asiaticoside Extraction and Chromatographic Analysis

Rationale for dilute acid extraction. Dilute hydrochloric acid was employed as the extraction solvent to disrupt plant cell walls and inactivate endogenous glycosidases, thereby facilitating the release of asiaticoside while minimising enzymatic degradation. The acid concentration was kept below 0.08 mol L^−1^ to avoid hydrolysis of the glycosidic bonds of the target saponin. The integrity of the extracted asiaticoside was confirmed by HPLC comparison with the reference standard. A stock solution of asiaticoside was prepared at 0.6 mg mL^−1^ in methanol for both extraction optimisation and HPLC calibration.

Extraction procedure. *C. asiatica* powder (3.00 g) was refluxed in a 250 mL round-bottom flask with HCl solution under the conditions specified below. After filtration, the filtrate was neutralised with 3–5% (*w*/*v*) sodium hydroxide solution, transferred to a 100 mL volumetric flask, diluted to volume with ultrapure water, and filtered through a 0.22 μm membrane for HPLC analysis.

HPLC analysis. Quantification was performed on an Agilent 1260 Infinity II HPLC system (Agilent Technologies, Santa Clara, CA, USA) equipped with a Shimadzu InertSustain C_18_ column (4.6 × 250 mm, 5 μm; Shimadzu, Kyoto, Japan). The mobile phase was methanol–acetonitrile–water (24:26:50, *v*/*v*/*v*) at a flow rate of 0.8 mL min^−1^. Detection was at 204 nm, injection volume 10 μL, and column temperature 30 °C. Quantification was performed by the external standard method. Test solutions of dried fractions were prepared at a theoretical concentration of 0.8 mg mL^−1^ in methanol and filtered through 0.22 μm membranes prior to injection. The asiaticoside content was calculated using the formula:

Content (%) = (A_sample_ × C_standard_ × 0.98)/(A_standard_ × C_sample_) × 100% (1)

where A_sample_ and A_standard_ are the peak areas of the sample and standard, C_sample_ is the theoretical concentration of the sample solution, C_standard_ is the concentration of the standard solution, and 0.98 is the purity correction factor of the reference standard. The HPLC method was validated for linearity (*R*^2^ > 0.999), precision (relative standard deviation < 2%), and recovery (98–102%).

Single-factor experiments. Four extraction parameters were evaluated by varying one factor while keeping the others constant: (i) HCl concentration: 0.01, 0.02, 0.03, 0.06, and 0.08 mol L^−1^ (solid–liquid ratio 1:30, 2 h, 1 cycle); (ii) solid–liquid ratio: 1:5, 1:8, 1:10, 1:15, and 1:30 (HCl 0.02 mol L^−1^, 2 h, 1 cycle); (iii) extraction time: 1.0, 1.5, 2.0, 2.5, and 3.0 h (HCl 0.02 mol L^−1^, ratio 1:15, 1 cycle); (iv) number of cycles: 1, 2, and 3 (HCl 0.02 mol L^−1^, ratio 1:15, 2 h per cycle).

Orthogonal optimisation. An L_9_(3^3^) orthogonal array was employed with three factors: A (HCl concentration, 0.01, 0.02, 0.03 mol L^−1^), B (solid–liquid ratio, 1:10, 1:15, 1:30), and C (extraction time, 1, 2, 3 h), with the number of cycles fixed at 2 (Table 1). The response variable was asiaticoside content.

### 3.3. Enrichment by Polyamide Column Chromatography

Polyamide (100 mL bed volume, BV) was activated sequentially by soaking in 95% ethanol, washing with ultrapure water, treating with 5% (*w*/*v*) NaOH for 4 h, washing with water, treating with 10% (*v*/*v*) acetic acid for 3.5 h, and finally washing with water to neutrality. The activated polyamide was slurry-packed into a glass column (100 mL bed volume) and equilibrated with ultrapure water. The optimised extract (Section 3.2) was loaded at a flow rate of 0.5 BV h^−1^. After washing with water until the eluate was colourless, stepwise elution was performed with 4 BV of 30% ethanol, 1 BV of 50% ethanol, and 1 BV of 95% ethanol. Fractions (25 mL each) were collected and monitored by HPLC. Target fractions containing asiaticoside were combined, concentrated under reduced pressure, and vacuum-dried.

### 3.4. Purification by Reversed-Phase C_18_ Column Chromatography and Structural Characterisation

UniSil30–120 C_18_ reversed-phase silica gel (Suzhou Nanomicro Technology Co., Ltd., Suzhou, China) was soaked in methanol overnight, then slurry-packed into a column (30 mL bed volume) using a VSP-2050 medium-pressure chromatography system (YAMAZEN, Osaka, Japan). The packed column was equilibrated with ultrapure water at a flow rate of 1 BV h^−1^. The enriched extract (1.000 g, Section 3.3) was dissolved in ultrapure water (0.1 g mL^−1^) by ultrasonication, loaded onto the column at 1 BV h^−1^, and washed with 2 BV of water. Gradient elution was performed with 10%, 30%, 50%, 70%, and 100% (*v*/*v*) methanol in water (1 BV per step), and fractions were monitored by HPLC. Fractions containing asiaticoside were combined, concentrated under reduced pressure, and vacuum-dried to afford a white amorphous powder.

The purified compound was characterised by HR-ESI-MS and NMR spectroscopy. HR-ESI-MS was performed on a Waters Xevo G3 QTOF mass spectrometer (Waters Corporation, Milford, MA, USA) in positive and negative ion modes. The molecular formula was confirmed by accurate mass measurement of the [M + Na]^+^ and [M + HCOO]^−^ adduct ions. ^1^H and ^13^C NMR spectra were recorded in methanol-d_4_ on a Bruker AVANCE NEO 600 NMR Spectrometer (Bruker Corporation, Billerica, MA, USA). The NMR data were compared with literature values for asiaticoside [39].

### 3.5. Molecular Docking

Crystal structures of SIRT1, HMGB1, p53, p21, IL-1β, TNF-α, and IL-6 were retrieved from the RCSB PDB. Proteins were preprocessed in PyMOL (version 3.1.8) by removing water, heteroatoms, and co-ligands. Polar hydrogens and Kollman charges were added in AutoDock Tools (version 1.5.7). The asiaticoside structure (PubChem CID: 11954067) was energy-minimised (MMFF94), assigned Gasteiger charges, and saved in PDBQT format. Docking used AutoDock Vina (version 1.2.3) with grid boxes centred on active sites (exhaustiveness 8). Top-ranked poses were analysed in UCSF Chimera (version 1.2) and PyMOL; 2D diagrams were from Discovery Studio Visualizer (version 4.0).

### 3.6. Cell Culture and Drug Treatment

HUVECs were obtained from FuHeng Cell Center (Shanghai, China). Cells were cultured in endothelial cell medium (ECM; ScienCell, cat no. 1001) supplemented with 10% (*v*/*v*) FBS, 100 U mL^−1^ penicillin, and 100 μg mL^−1^ streptomycin, at 37 °C in a humidified atmosphere containing 5% CO_2_. The medium was changed every 2–3 days, and cells between passages 3 and 8 were used for all experiments. As the cells were commercially obtained and anonymised, no additional ethical approval was required.

Preparation of H_2_O_2_ and drugs. H_2_O_2_ (30% *w*/*w*) was diluted in serum-free ECM immediately before each experiment to achieve the desired concentration. Based on preliminary dose-finding experiments and previous reports, 400 μM H_2_O_2_ for 4 h was selected as the senescence-inducing condition, as it produced significant senescence markers without causing complete cell death. Asiaticoside and resveratrol were dissolved in DMSO to prepare stock solutions and diluted in culture medium to the indicated concentrations (final DMSO concentration ≤ 0.1% *v*/*v* in all groups).

General treatment protocol. HUVECs were pretreated with asiaticoside (10, 30, or 100 μM) or resveratrol (10 μM) for 24 h, then exposed to 400 μM H_2_O_2_ in serum-free ECM for 4 h, followed by 24 h recovery in complete ECM. For mechanistic experiments (Section 3.10, Section 3.11 and Section 3.12), HUVECs seeded in 12-well plates (1 × 10^5^ cells per well) were pretreated with 100 μM asiaticoside or vehicle for 24 h, exposed to 400 μM H_2_O_2_ in serum-free ECM for 4 h, and then recovered in complete ECM for 24 h. Based on the dose–response results of SA-β-gal staining (Section 3.8), 100 μM was selected as the optimal protective concentration. Experimental groups were: control (serum-free ECM without H_2_O_2_), model (400 μM H_2_O_2_), asiaticoside (100 μM asiaticoside + 400 μM H_2_O_2_), and resveratrol (10 μM resveratrol + 400 μM H_2_O_2_, positive control).

### 3.7. Cell Viability Assay (MTT)

To determine the non-toxic concentration range of asiaticoside and resveratrol, HUVECs were seeded in 96-well plates at a density of 5 × 10^3^ cells per well and cultured for 24 h. Cells were then treated with asiaticoside (10, 30, or 100 μM) or resveratrol (10 μM) for 24 h. 3-(4,5-Dimethylthiazol-2-yl)-2,5-diphenyltetrazolium bromide (MTT; Beyotime Biotechnology, Shanghai, China) was dissolved in phosphate-buffered saline (PBS) at 5 mg mL^−1^ and added to each well at a final concentration of 0.5 mg mL^−1^. After incubation for 4 h at 37 °C, the medium was removed, and the resulting formazan crystals were dissolved in DMSO. Absorbance was measured at 570 nm using a Cytation 1 multi-mode microplate reader (BioTek Instruments, Winooski, VT, USA). Five replicate wells per condition; three independent experiments. Cell viability was expressed as a percentage of the vehicle control.

### 3.8. Senescence-Associated β-Galactosidase (SA-β-Gal) Staining

HUVECs were seeded in 48-well plates at a density of 1 × 10^4^ cells per well and subjected to the treatment protocol described in Section 3.6. Senescence-associated β-galactosidase (SA-β-gal) activity was detected using a SA-β-gal Staining Kit (Beyotime Biotechnology) according to the manufacturer’s instructions. Briefly, cells were fixed with the fixation buffer for 15 min at room temperature, washed with PBS, and incubated with the X-gal staining solution at 37 °C for 12–16 h in the dark without CO_2_. SA-β-gal-positive cells (stained blue) were counted in three randomly selected fields per well under a light microscope. Three replicate wells per condition; three independent experiments. Results were quantified using ImageJ (version 1.8.0, National Institutes of Health, Bethesda, MD, USA) and expressed as the percentage of SA-β-gal-positive cells relative to the total cell count.

### 3.9. Cell Cycle Analysis

After treatment as described in Section 3.6, cells were harvested by trypsinisation with 0.25% trypsin without EDTA (Shanghai Fuheng Biotechnology Co., Ltd., Shanghai, China), washed with cold PBS, and fixed in 70% (*v*/*v*) ethanol at 4 °C overnight. Fixed cells were washed with PBS, resuspended in 200 μL of PBS containing 50 μL propidium iodide and 20 μL RNase A (Merck, Darmstadt, Germany), and incubated at room temperature for 30 min in the dark. DNA content was measured on a CytoFLEX flow cytometer (Beckman Coulter, Brea, CA, USA), and cell cycle distribution was analysed using CytExpert software (version 3.6, Beckman Coulter).

### 3.10. Enzyme-Linked Immunosorbent Assay (ELISA)

Culture supernatants were collected after treatment (Section 3.6), centrifuged at 1500× *g* for 10 min at 4 °C to remove cellular debris, and stored at −80 °C until analysis. The concentrations of interleukin-6 (IL-6), tumour necrosis factor-α (TNF-α), interleukin-8 (IL-8), high mobility group box 1 (HMGB1), and interleukin-1β (IL-1β) in the supernatants were measured using human ELISA kits (BYabscience, Shanghai, China; cat. nos. BY-EH110629, BY-EH110436, BY-EH110351, BY-EH111776, and BY-EH110377, respectively) according to the manufacturer’s instructions. Absorbance was read at 450 nm using Cytation 1. All samples were measured in triplicate.

### 3.11. Quantitative Real-Time Polymerase Chain Reaction (qPCR)

Total RNA was extracted using the RNAprep Pure Cell/Bacteria Kit (TIANGEN Biotech, Beijing, China). RNA purity was assessed by the A_260_/A_280_ ratio using Cytation 1, and integrity was verified by 1% agarose gel electrophoresis. Complementary DNA (cDNA) was synthesised from 1 μg of total RNA using the FastQuant RT Kit with gDNase (TIANGEN) to remove genomic DNA contamination. Quantitative real-time polymerase chain reaction (qPCR) was performed using FastReal qPCR PreMix II (SYBR Green; TIANGEN) in 20 μL reactions on a QuantStudio 3 Real-Time PCR System (Applied Biosystems, Foster City, CA, USA) under the following conditions: 95 °C for 2 min, followed by 40 cycles of 95 °C for 5 s and 60 °C for 30 s. Primer sequences for IL-1β, TNF-α, HMGB1, p21, p16, SIRT1, and glyceraldehyde-3-phosphate dehydrogenase (GAPDH) are listed in Table S1 (Supplementary Information). Relative gene expression was calculated by the 2^−ΔΔ^Ct method with GAPDH as the internal reference.

### 3.12. Western Blot Analysis

After treatment (Section 3.6), cells were lysed in radioimmunoprecipitation assay (RIPA) buffer supplemented with protease and phosphatase inhibitors (Beyotime Biotechnology). Protein concentration was determined using a bicinchoninic acid (BCA) assay kit (Beyotime Biotechnology). Equal amounts of protein (20–30 μg) were separated by 10% sodium dodecyl sulfate–polyacrylamide gel electrophoresis (SDS-PAGE; 80 V stacking, 120 V resolving) and transferred onto polyvinylidene difluoride (PVDF) membranes (0.45 μm; Merck Millipore) by wet transfer at 200 mA for 90 min on ice. Membranes were blocked with 5% (*w*/*v*) non-fat milk in Tris-buffered saline with 0.1% Tween-20 (TBST) for 1 h at room temperature.

Primary antibodies were incubated overnight at 4 °C in 5% BSA/TBST or 5% milk/TBST: anti-GAPDH (1:5000, cat. 60004-1-Ig, mouse monoclonal), anti-SIRT1 (1:1000, cat. 13161-1-AP, rabbit polyclonal), anti-HMGB1 (1:1000, cat. 10829-1-AP, rabbit polyclonal), anti-p53 (1:2000, cat. 60283-2-Ig, mouse monoclonal), and anti-p21 (1:1000, cat. 10355-1-AP, rabbit polyclonal) (all from Proteintech, Wuhan, China). After washing with TBST (3 × 10 min), membranes were incubated with horseradish peroxidase (HRP)-conjugated secondary antibodies (1:5000; anti-mouse IgG, cat. SA00001-1, for GAPDH and p53; anti-rabbit IgG, cat. SA00001-2, for SIRT1, HMGB1, and p21; Proteintech) for 1 h at room temperature. After washing (3 × 10 min), bands were visualised by enhanced chemiluminescence (ECL; Beyotime Biotechnology) and imaged on a Tanon 5200 chemiluminescence imaging system (Shanghai Tianneng Technology Co., Ltd., Shanghai, China). Band intensities were quantified using ImageJ and normalised to GAPDH.

### 3.13. Statistical Analysis

All data are presented as mean ± standard deviation (SD) from at least three independent experiments. Normality of data distribution was assessed by the Shapiro–Wilk test, and homogeneity of variance was evaluated by Levene’s test. For comparisons among three or more groups, one-way analysis of variance (ANOVA) followed by Tukey’s post hoc test was used. For comparisons between two groups, unpaired Student’s *t*-test was applied. Statistical analyses were performed using GraphPad Prism 7 (GraphPad Software, now Dotmatics, San Diego, CA, USA). A *p*-value < 0.05 was considered statistically significant. Significance levels are denoted as * *p* < 0.05, ** *p* < 0.01.

## 4. Conclusions

In summary, this study established a green integrated strategy combining acid hydrolysis extraction, polyamide enrichment, and C_18_ reversed-phase purification, which efficiently yielded asiaticoside with a purity exceeding 98% from *Centella asiatica*. The use of dilute HCl as the primary extraction solvent substantially reduced organic solvent consumption, aligning with the principles of green chemistry. The high-purity asiaticoside obtained through this process was directly used in subsequent cell-based assays, where it demonstrated significant anti-senescence activity. Molecular docking-guided target fishing revealed that asiaticoside possesses a polypharmacological binding profile, with the potential to directly interact with SIRT1, the HMGB1–DNA complex, TNF-α, IL-1β, IL-6, p53, and p21—key nodes of the senescence regulatory network. Cellular validation in H_2_O_2_-induced senescent HUVECs demonstrated that asiaticoside, particularly at 100 μM, significantly attenuated SA-β-gal activity and cell cycle arrest without compromising cell viability. Mechanistically, asiaticoside restored SIRT1 expression, suppressed the secretion of multiple SASP components (IL-1β, IL-6, IL-8, TNF-α, and HMGB1), downregulated p21 at both mRNA and protein levels, reduced p53 protein expression, and suppressed p16 mRNA expression. These findings suggest that asiaticoside may mitigate endothelial senescence and SASP-driven inflammation by modulating the SIRT1/HMGB1/p53–p21 axis, although direct functional validation is still required. Future work should include SIRT1 deacetylase activity assays, siRNA- or CRISPR-based knockdown of SIRT1/HMGB1, direct binding assays such as SPR or CETSA, and in vivo models of vascular ageing to confirm the proposed mechanism.

## Figures and Tables

**Figure 1:**
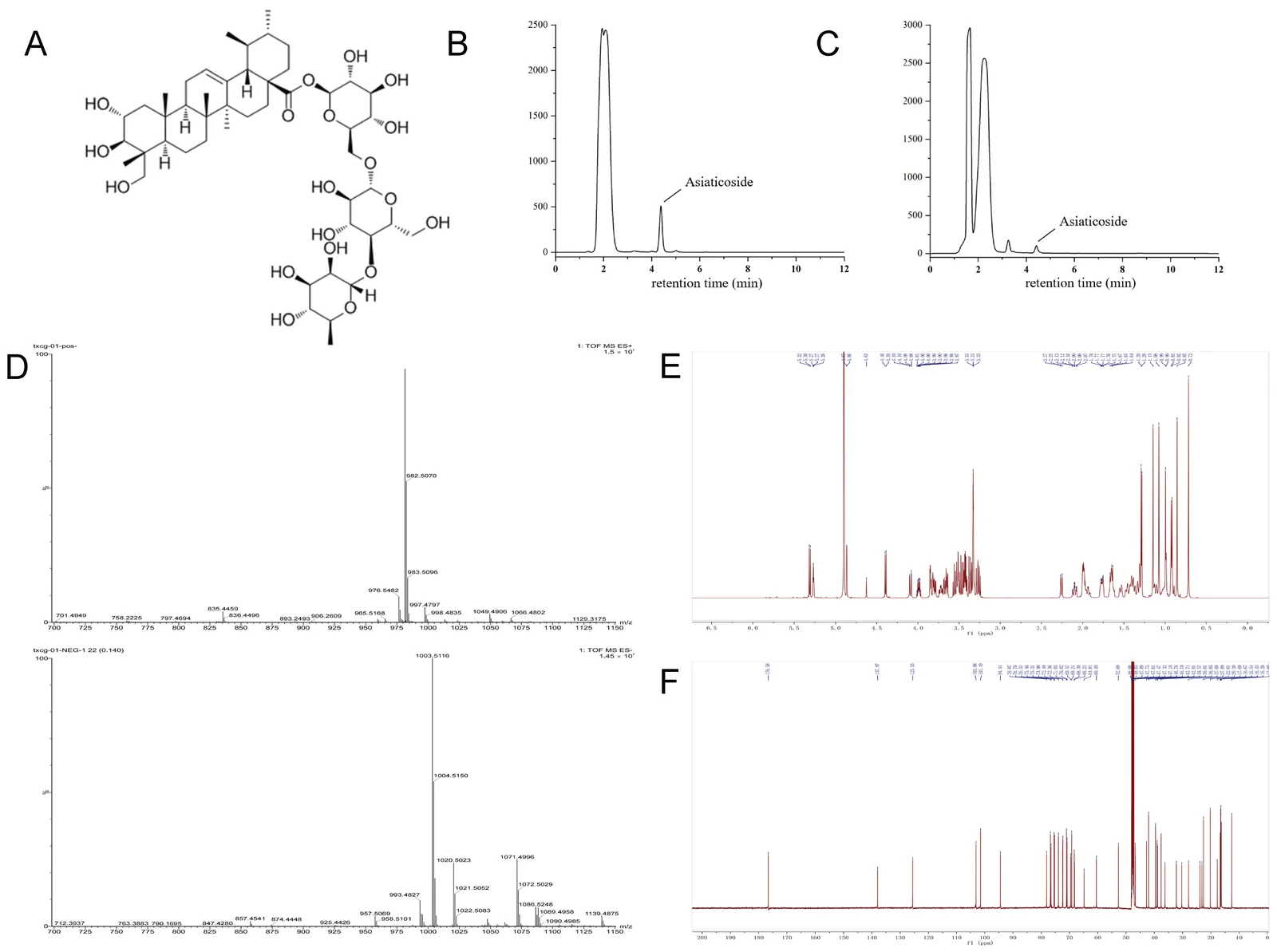
Structural characterisation of asiaticoside. (**A**) Chemical structure of asiaticoside. (**B**) HPLC chromatogram of the asiaticoside reference standard. (**C**) HPLC chromatogram of the asiaticoside sample obtained from the extraction. (**D**) HR-ESI-MS spectrum of asiaticoside. (**E**) ^1^H NMR spectrum of asiaticoside (600 MHz, methanol-d_4_). (**F**) ^13^C NMR spectrum of asiaticoside (600 MHz, methanol-d_4_).

**Figure 2:**
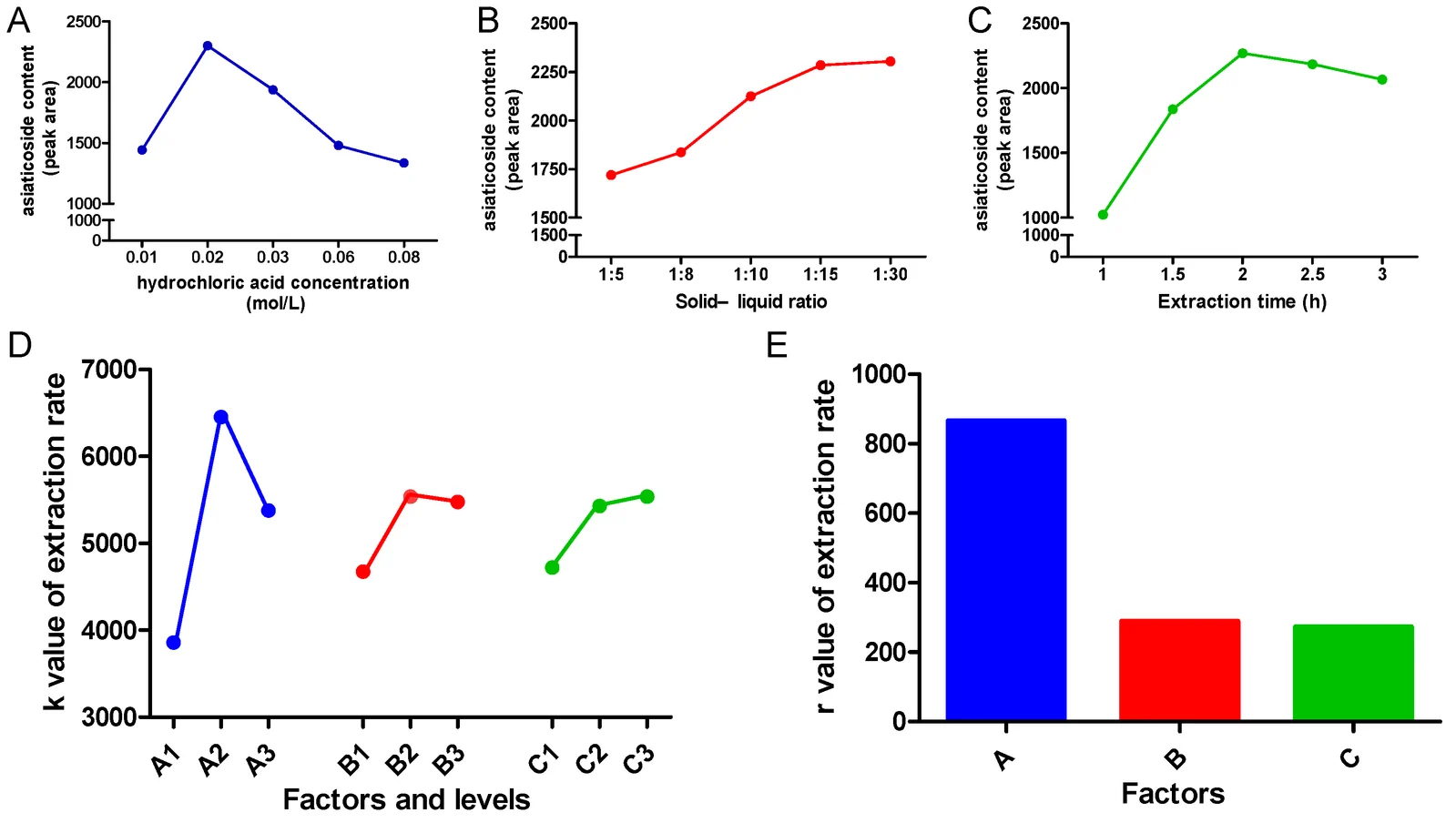
Optimisation of asiaticoside extraction. (**A**–**C**) Single-factor experiments showing the effects of (**A**) HCl concentration, (**B**) solid–liquid ratio, and (**C**) extraction time on the asiaticoside peak area. (**D**) *k* value curves from the L_9_(3^3^) orthogonal experiment. (**E**) Range (*R*) comparison of the three factors. Data are expressed as mean ± SD (*n* = 3).

**Figure 3:**
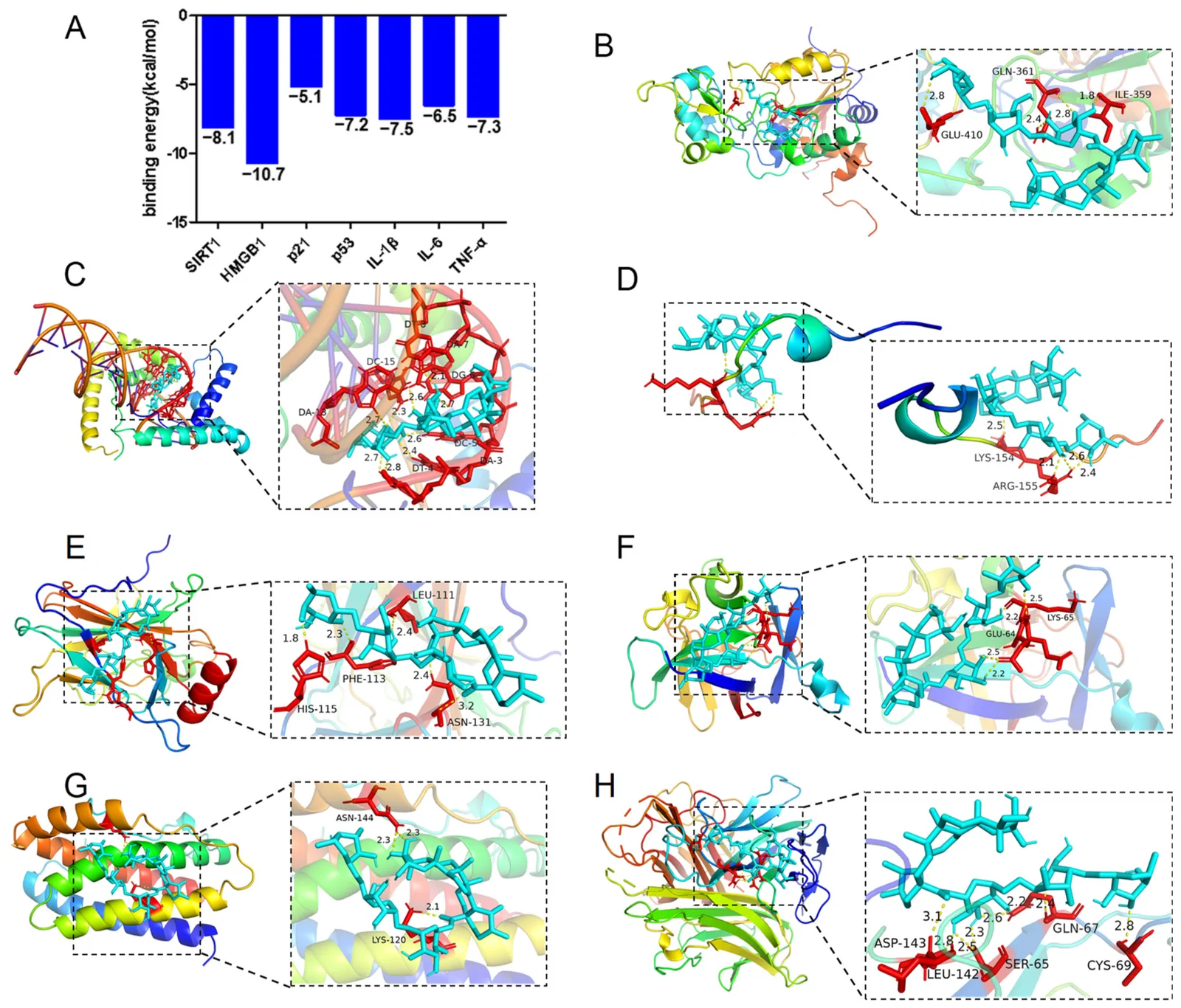
Molecular docking analysis of asiaticoside with senescence- and inflammation-related target proteins. (**A**) Binding affinities (kcal mol^−1^) of asiaticoside toward SIRT1, HMGB1 (DNA complex), TNF-α, IL-1β, IL-6, p53, and p21 predicted by AutoDock Vina. More negative values indicate stronger predicted binding. (**B**–**H**) Overall binding modes (**left panels**) and enlarged views of key interacting residues (**right panels**) for asiaticoside docked into (**B**) SIRT1 (PDB ID: 4KXQ), (**C**) the HMGB1–DNA complex (PDB ID: 4QR9), (**D**) TNF-α(PDB ID: 2E7A), (**E**) IL-1β(PDB ID: 1I1B), (**F**) IL-6(PDB ID: 1ALU), (**G**) p53 (PDB ID: 1TUP), and (**H**) p21(PDB ID: 1AXC). Asiaticoside is shown as cyan sticks, key interacting residues as red sticks, and hydrogen bonds as yellow dashed lines with donor–acceptor (heavy-atom) distances labelled in Å.

**Figure 4:**
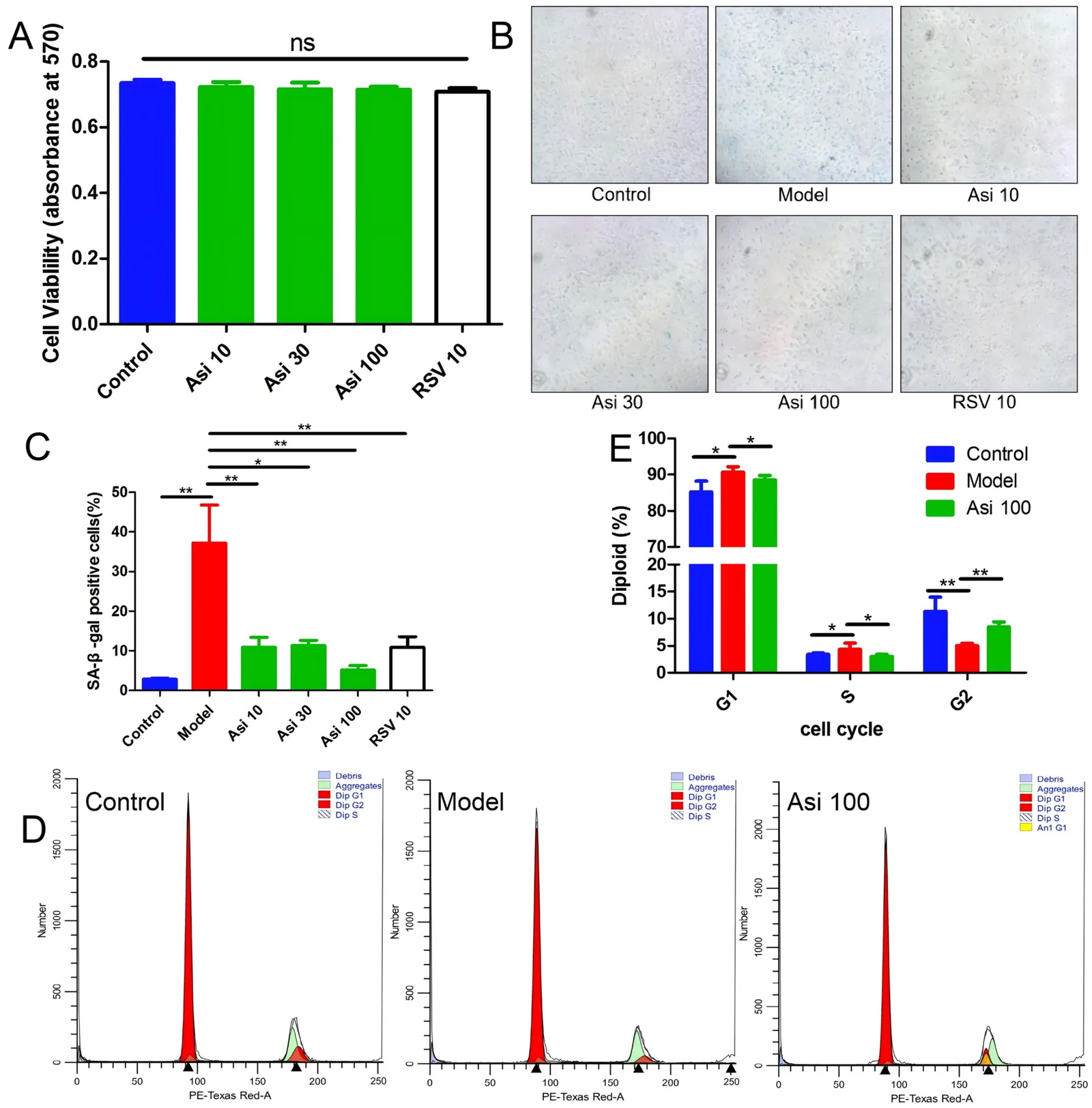
Asiaticoside attenuates H_2_O_2_-induced cytotoxicity, senescence, and cell cycle arrest in HUVECs. (**A**) Cell viability determined by the MTT assay after treatment with asiaticoside (10, 30, and 100 μM) or resveratrol (10 μM) for 24 h. Data are presented as mean ± SD (*n* = 5). (**B**) Representative micrographs of SA-β-gal staining in the control, H_2_O_2_ model (400 μM), H_2_O_2_ + asiaticoside (10, 30, and 100 μM), and H_2_O_2_ + resveratrol (10 μM) groups. Images were captured at 200× magnification. (**C**) Quantification of SA-β-gal-positive cells. Data are mean ± SD (*n* = 3). (**D**) Representative flow cytometry histograms showing cell cycle distribution determined by propidium iodide staining. (**E**) Percentages of cells in the G0/G1, S, and G2/M phases for each treatment group. Data are mean ± SD (*n* = 3). * *p* < 0.05, ** *p* < 0.01, ns not significant. Asi, asiaticoside; RSV, resveratrol.

**Figure 5:**
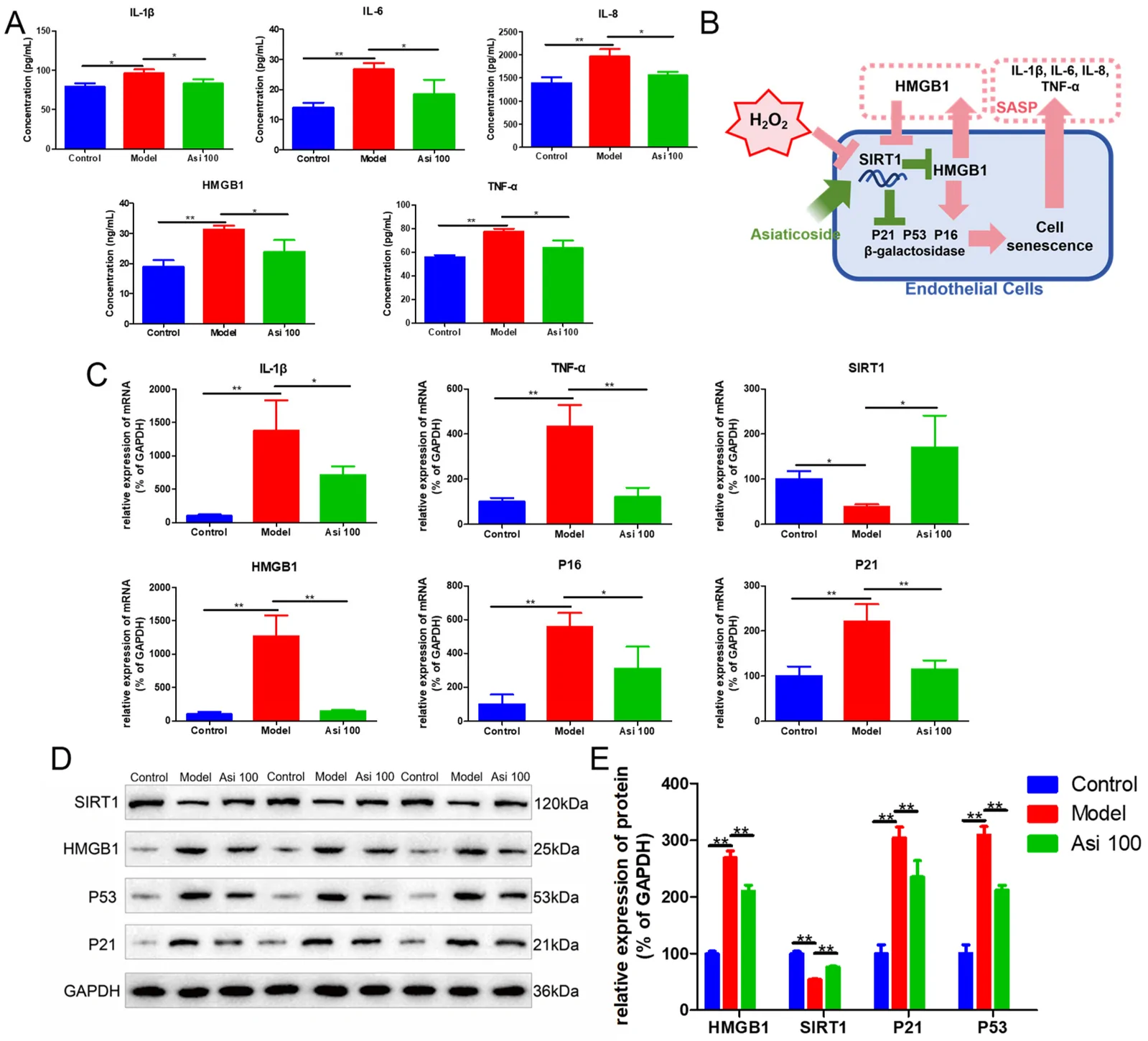
Asiaticoside suppresses the senescence-associated secretory phenotype and modulates the SIRT1/HMGB1/p53–p21 pathway in H_2_O_2_-treated HUVECs. (**A**) ELISA quantification of secreted IL-1β, IL-6, IL-8, TNF-α, and HMGB1 in the culture supernatants. Data are mean ± SD (*n* = 3 independent experiments). (**B**) Schematic illustration of the proposed mechanism by which asiaticoside attenuates endothelial senescence through the SIRT1/HMGB1 axis. (**C**) qPCR analysis of *IL1B*, *TNF*, *CDKN1A (p21)*, *CDKN2A (p16)*, *SIRT1*, and *HMGB1* mRNA expression, normalised to GAPDH. Data are mean ± SD (*n* = 3). (**D**) Western blot bands of HMGB1, SIRT1, p21, p53, and GAPDH (loading control). The cropped blot images shown in (**D**) were obtained from the full blots presented in Figures S1–S5 (Supplementary Information). (**E**) Densitometric quantification of HMGB1, SIRT1, p21, and p53 protein levels relative to GAPDH. Data are mean ± SD (*n* = 3). * *p* < 0.05, ** *p* < 0.01. Treatment groups: control (0.1% DMSO), H_2_O_2_ model (400 μM), and H_2_O_2_ + asiaticoside (100 μM). Asi, asiaticoside.

**Table 1:** Factors and levels for L_9_(3^3^) orthogonal experiment.

Level	A (HCl Concentration, mol L^−1^)	B (Solid–Liquid Ratio)	C (Extraction Time, h)
1	0.01	1:10	1
2	0.02	1:15	2
3	0.03	1:30	3

**Table 2:** Orthogonal test design and results.

Run	A	B	C	Asiaticoside Peak Area
1	1	1	1	856.321
2	1	2	3	1393.977
3	1	3	2	1606.708
4	2	1	3	2168.499
5	2	2	2	2282.135
6	2	3	1	2003.758
7	3	1	2	1649.685
8	3	2	1	1861.937
9	3	3	3	1868.061
k_1_	3857.01	4674.5	4722.02	
k_2_	6454.39	5538.05	5430.54	
k_3_	5379.68	5478.53	5538.53	
R	865.80	287.85	272.17	

Note: k_1_, k_2_ and k_3_ are the sum of the peak areas at each level for each factor; R is the range.

**Table 3:** Analysis of variance.

Source of Variance	Sum of Squares	Degrees of Freedom	Mean Square	*F* Value	*p* Value
A	1,135,550.968	2	567,775.484	13.679	0.068
B	155,078.054	2	77,539.027	1.868	0.349
C	131,150.657	2	65,575.328	1.58	0.388

Note: *R*^2^ = 0.945.

## Data Availability

The original contributions presented in this study are included in the article/Supplementary Materials. Further inquiries can be directed to the corresponding author.

## Supplementary Materials

The following supporting information can be downloaded at: https://www.mdpi.com/article/10.3390/molecules31193451/s1, Figure S1. Full blot images of GAPDH. (A) Original uncropped blot image of GAPDH. (B) The same blot with lane annotations indicating sample identities. The nearest molecular weight marker band is labeled on the left. Figure S2. Full blot images of p21. (A) Original uncropped blot image of p21. (B) The same blot with lane annotations indicating sample identities. The nearest molecular weight marker band is labeled on the left. Figure S3. Full blot images of p53. (A) Original uncropped blot image of p53. (B) The same blot with lane annotations indicating sample identities. The nearest molecular weight marker band is labeled on the left. Figure S4. Full blot images of HMGB1. (A) Original uncropped blot image of HMGB1. (B) The same blot with lane annotations indicating sample identities. The nearest molecular weight marker band is labeled on the left. Figure S5. Full blot images of SIRT1. (A) Original uncropped blot image of SIRT1. (B) The same blot with lane annotations indicating sample identities. The nearest molecular weight marker band is labeled on the left. Figure S6. Representative SA-β-gal staining images from three independent experiments. HUVECs were treated as described in Section 3.8. Images were captured at 200× magnification. Table S1. Primer sequences for qPCR.
