# Supplementary material for: Green Extraction and Molecular Docking-Guided Discovery of Asiaticoside as a Multi-Target Modulator of the SIRT1/HMGB1/p53-p21 Axis in H2O2-Induced Senescent Endothelial Cells
Source: Molecules. 2026 Sep 28;31(19):3451. doi: 10.3390/molecules31193451 (PMC13666185; doi:10.3390/molecules31193451)
Supplement: Supplementary file 1 [file molecules-31-03451-s001.zip › molecules-4546457-supplementary.pdf]

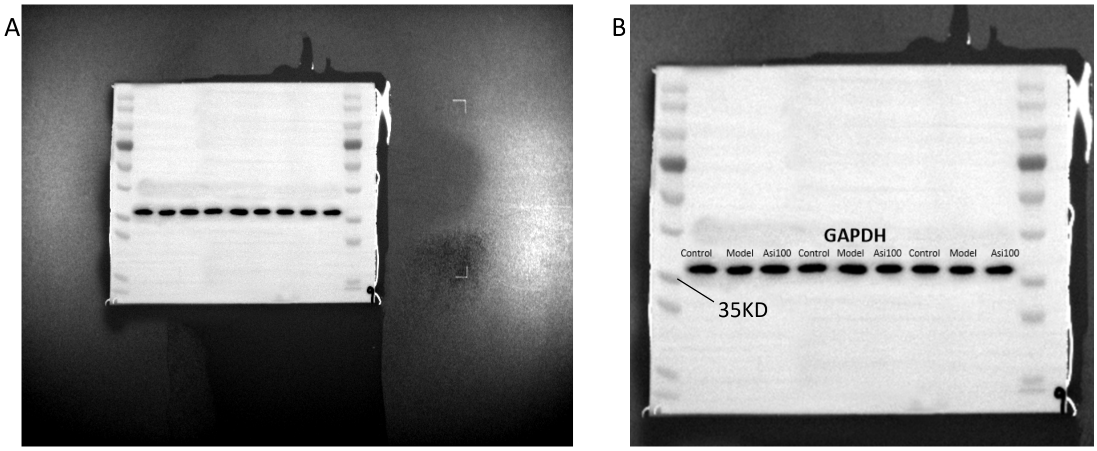

Figure S1 Full blot images of GAPDH. (A) Original uncropped blot image of GAPDH. (B) The same blot with lane annotations indicating sample identities. The nearest molecular weight marker band is labeled on the left.

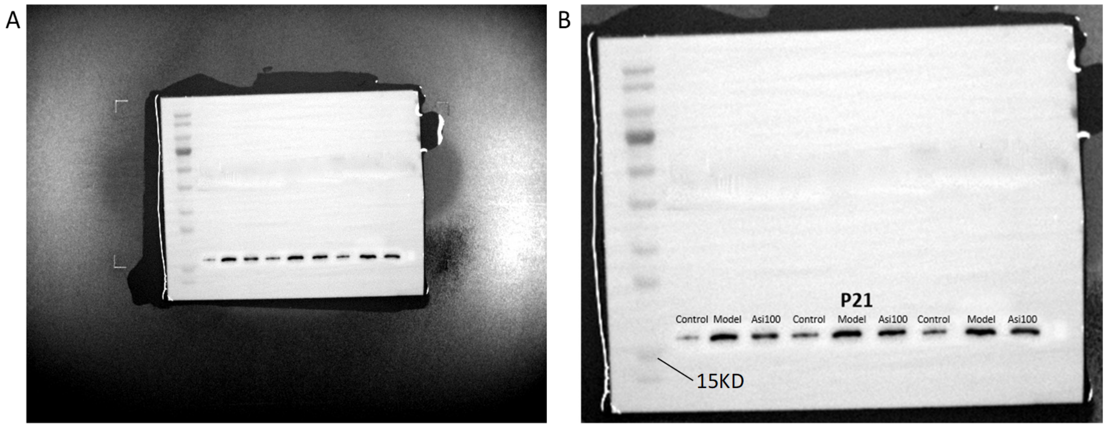

Figure S2 Full blot images of p21. (A) Original uncropped blot image of p21. (B) The same blot with lane annotations indicating sample identities. The nearest molecular weight marker band is labeled on the left.

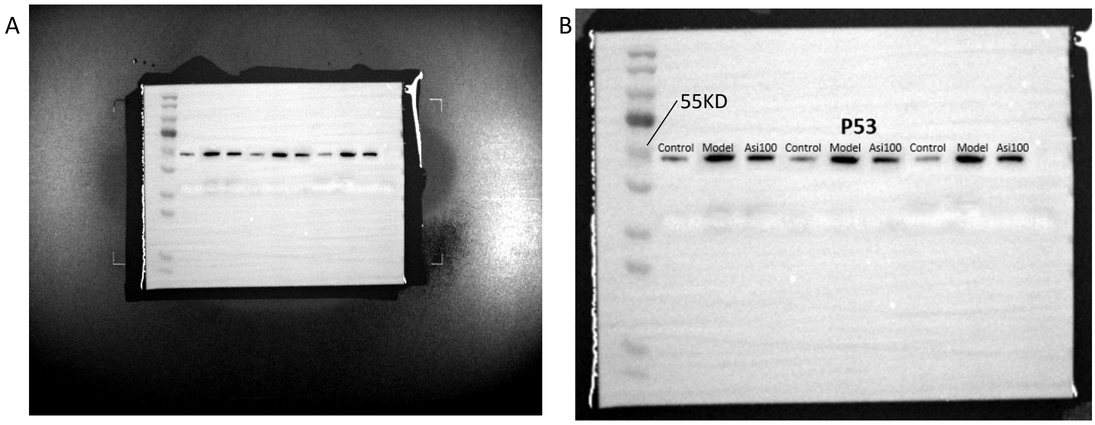

Figure S3 Full blot images of p53. (A) Original uncropped blot image of p53. (B) The same blot with lane annotations indicating sample identities. The nearest molecular weight marker band is labeled on the left.

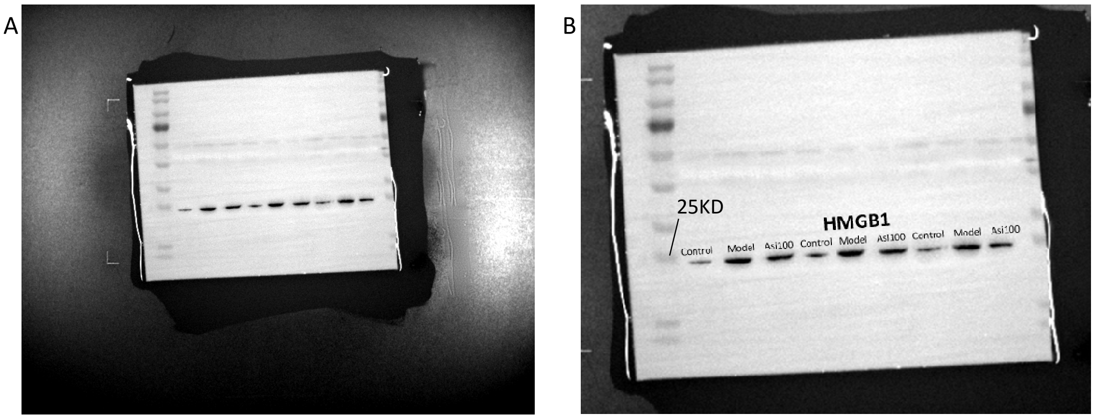

Figure S4 Full blot images of HMGB1. (A) Original uncropped blot image of HMGB1. (B) The same blot with lane annotations indicating sample identities. The nearest molecular weight marker band is labeled on the left.

A B

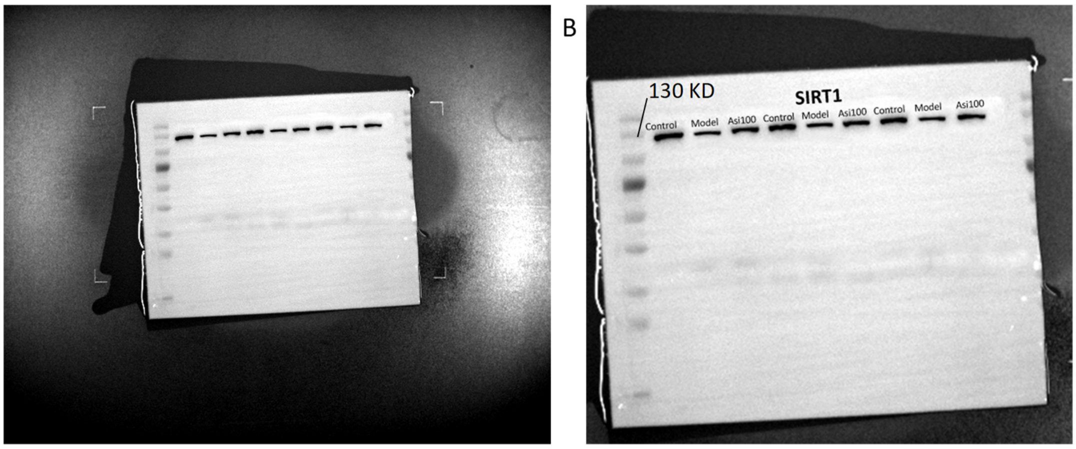

Figure S5 Full blot images of SIRT1. (A) Original uncropped blot image of SIRT1. (B) The same blot with lane annotations indicating sample identities. The nearest molecular weight marker band is labeled on the left.

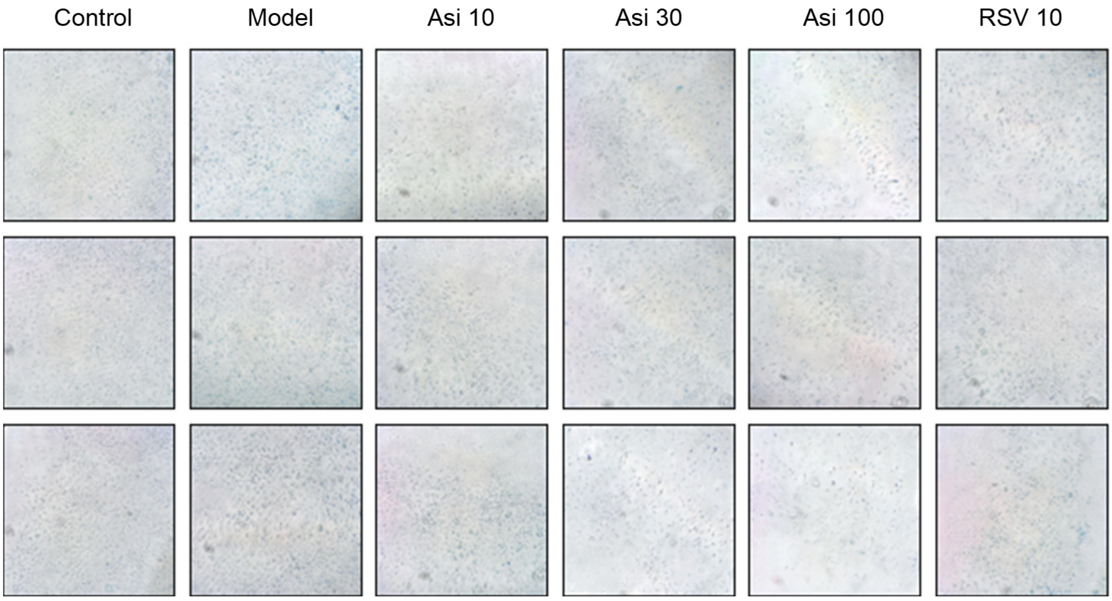

Figure S6 Representative SA-β-gal staining images from three independent experiments. HUVECs were treated as described in Section 3.8. Images were captured at 200× magnification.

Table S1 Primer sequences for qPCR

Gene	Forward primer (5′→3′)	Reverse primer (5′→3′)
IL1B	CGAATCTCCGACCACCACTA	AGCCTCGTTATCCCATGTGT
TNF	TCAGAGGGCCTGTACCTCAT	GGAAGACCCCTCCCAGATAG
HMGB1	GCGAAGAAACTGGGAGAGATGTG	GCATCAGGCTTTCCTTTAGCTCG
CDKN1A (p21)	CACCGAGACACCACTGGAG	AGGCACAAGGGTACAAGACA
CDKN2A (p16)	ACTCTCACCCGACCCGTG	CATGGTTACTGCCTCTGGTG
SIRT1	AGCAGATTAGTAGGCGGCTT	GACTCTGGCATGTCCCACTA
GAPDH	TCAAGAAGGTGGTGAAGCAGG	TCAAAGGTGGAGGAGTGGGT
